# Dealing of mass casualty in ophthalmology - A challenge

**DOI:** 10.4103/0974-620X.64243

**Published:** 2010

**Authors:** Kalpana Pandey, Parul Singh

**Affiliations:** Department of Ophthalmology, Government Medical College, Haldwani, Nainital, Uttarakhand, India

Sir,

When immediate management in mass cases becomes need of the hour it becomes a challenge even to the most experienced of clinicians. Since ophthalmologists seldom get chance to deal such mass emergencies, it becomes all the more imperative to be prepared. One such challenging situation was encountered on 15^th^April 08 by eye surgeons of UFHT hospital. Nineteen cases of chemical burn were admitted in the casualty department.

There is usually a time interval between the hospital being informed and patients being actually brought in. This is the golden time to activate the hospital emergency plan so that maximum capacity regarding medical personnel, equipments and pharmaceuticals is mobilized. The emergency department (ED) should be immediately cleared of the stable patients. If the ED staff already knows the chain of command, at the time of mass casualty incidence (MCI) there is better and faster coordination, communication and use of harmonized nomenclature.

This was an incidence of ocular mass casualty in particular i.e. when the injury is solely of the ocular type. The ED team is better trained in dealing with general life threatening mass casualties rather than of any particular specialty. The result was that they could not be of much help to the ophthalmic staff. Moreover the ophthalmic staff rarely witnesses an ocular mass casualty. Ophthalmologists are usually called for ophthalmic management as a part of mass casualty. Consequently a command line was missing and there was not a proper coordination at least for the initial few minutes. Since this was the incidence of chemical burn, initial time was most crucial and having lost even a single minute might have affected the final visual outcome.

A quick work distribution was done among the eye surgeons and the paramedical staff. Airway, breathing and circulation were checked and required management given. Simultaneously local anesthetic drops were instilled into the eyes and systemic painkillers given to the patients. Upper eye lid was everted and a vigorous wash with normal saline was done in each eye till the pH was found to be neutral. Whole cul-de-sac was explored with sterilized cotton buds for any loose necrosed tissue or embedded particulate matter.

Now with emergency treatment over, panic situation under control and patients shifted into eye ward, further management was planned. Painkillers and sedatives were given according to the agitation of the patients. Topical antibiotics, cycloplegics, steroids and antiglaucoma medication were started. Oral Vitamin A and Vitamin C supplement was also given. Grading of injury was done later on slit lamp and patients were categorized accordingly.

Four patients with grade IV and grade V injuries were referred to higher center for further management; remaining fifteen patients were managed and followed up in our hospital.

To conclude, the ophthalmic and ED staff should be well-trained in advance to work together under ocular mass-casualty incidence and regular drills about mass casualty management should be conducted. This would prevent confusion at the time of actual implementation.

